# Influenza Vaccination Implementation in Sri Lanka: A Cost-Effectiveness Analysis

**DOI:** 10.3390/vaccines11050932

**Published:** 2023-05-03

**Authors:** Coralei E. Neighbors, Evan R. Myers, Nayani P. Weerasinghe, Gaya B. Wijayaratne, Champica K. Bodinayake, Ajith Nagahawatte, L. Gayani Tillekeratne, Christopher W. Woods

**Affiliations:** 1 Hubert-Yeargan Center for Global Health, Duke University, Durham, NC 27710, USA; 2 Division of Women’s Community and Population Health, Department of Obstetrics & Gynecology, Duke University School of Medicine, Durham, NC 27710, USA; 3 Department of Microbiology, Faculty of Medicine, University of Ruhuna, Galle 80000, Sri Lanka; 4 Department of Medicine, Faculty of Medicine, University of Ruhuna, Galle 80000, Sri Lanka; 5 Duke Global Health Institute, Duke University, Durham, NC 27710, USA; 6 Division of Infectious Diseases, Department of Medicine, Duke University School of Medicine, Durham, NC 27710, USA

**Keywords:** cost-effectiveness, influenza, vaccination, Sri Lanka, Markov model, economic evaluation

## Abstract

Influenza causes an estimated 3 to 5 million cases of severe illness annually, along with substantial morbidity and mortality, particularly in low- and middle-income countries (LMICs). Currently, Sri Lanka has no influenza vaccination policies and does not offer vaccination within the public healthcare sector. Therefore, we performed a cost-effectiveness analysis of influenza vaccine implementation for the Sri Lankan population. We designed a static Markov model that followed a population cohort of Sri Lankans in three age groups, 0–4, 5–64, and 65+ years, through two potential scenarios: trivalent inactivated vaccination (TIV) and no TIV across twelve-monthly cycles using a governmental perspective at the national level. We also performed probabilistic and one-way sensitivity analyses to identify influential variables and account for uncertainty. The vaccination model arm reduced influenza outcomes by 20,710 cases, 438 hospitalizations, and 20 deaths compared to no vaccination in one year. Universal vaccination became cost-effective at approximately 98.01% of Sri Lanka’s 2022 GDP per capita (incremental cost-effectiveness ratio = 874,890.55 Rs/DALY averted; 3624.84 USD/DALY averted). Results were most sensitive to the vaccine coverage in the 5–64-year-old age group, the cost of the influenza vaccine dose in the 5–64-years-old age group, vaccine effectiveness in the under-5-years-old age group, and the vaccine coverage in the under-5-years-old age group. No value for a variable within our estimated ranges resulted in ICERs above Rs. 1,300,000 (USD 5386.15) per DALY adverted. Providing influenza vaccines was considered highly cost-effective compared to no vaccines. However, large-scale national studies with improved data are needed to better inform estimates and determine the impact of vaccination implementation.

## 1. Introduction

Influenza is an acute respiratory infectious disease that has been known to cause a substantial burden on public health systems globally [1]. Influenza viruses cause an estimated 3 to 5 million cases of severe illness annually [2], along with substantial morbidity and mortality, particularly in low- and middle-income countries (LMICs) [3]. In 2017, an estimated 297,000 influenza cases and 6000 hospitalizations occurred within Sri Lanka alone [4]. 

The most effective way to substantially decrease the annual burden from seasonal influenza epidemics is through equitable access to influenza vaccines [2]. Without readily available influenza vaccinations for all citizens, a country is missing a substantial disease prevention opportunity [5]. Influenza vaccines have been in use since 1936 and have since been integrated into numerous national immunization programs. As of 2016, seventy-four tropical and subtropical countries, representing 60% of the world’s population, did not have national vaccination policies regarding seasonal influenza and had a vaccine coverage rate of less than 5 per 1000 [6]. Sri Lanka currently does not routinely administer influenza vaccines in its public healthcare sector and does not include them in its National Immunization Programme (NIP) [6].

To implement a national influenza vaccination policy, policy makers must consider country-specific information. Factors to consider include seasonality and the disease burden within the country. In temperate regions, influenza is a seasonal disease that typically occurs in the winter months; however, tropical areas have no clearly defined seasonal patterns and can experience year-round influenza circulation with multiple peaks [2]. Even with differing seasonality in tropical and subtropical regions, vaccination has proven to be a cost-effective solution for reducing the influenza burden in tropical and subtropical regions [7,8]. 

For this study, we constructed a Markov model to determine the cost-effectiveness of influenza vaccinations for the Sri Lankan population from a societal perspective. Through this work, we hope to provide policy makers in Sri Lanka with valuable information for future decisions regarding influenza vaccine implementation in the Sri Lankan NIP. We also hope to provide information to researchers and policy makers in other data-limited LMICs and tropical settings to encourage the exploration of the cost-effectiveness of influenza vaccines in similar settings. 

## 2. Materials and Methods

### 2.1. Model Setting

Sri Lanka is a middle-income, tropical country that experiences year-round influenza cases with two peak circulation periods, occurring roughly between April to June and November to February [9,10,11,12]. Sri Lanka has a population of approximately 22.2 million people, of whom over 90.0% are under the age of 65 years [13]. As for healthcare, Sri Lanka offers publicly financed public healthcare free to all citizens [14]. The public healthcare sector makes up approximately 50% of outpatient services, 90% of inpatient admissions, and nearly all preventive services [14]. 

### 2.2. Model Design and Structure

**Figure 1:**
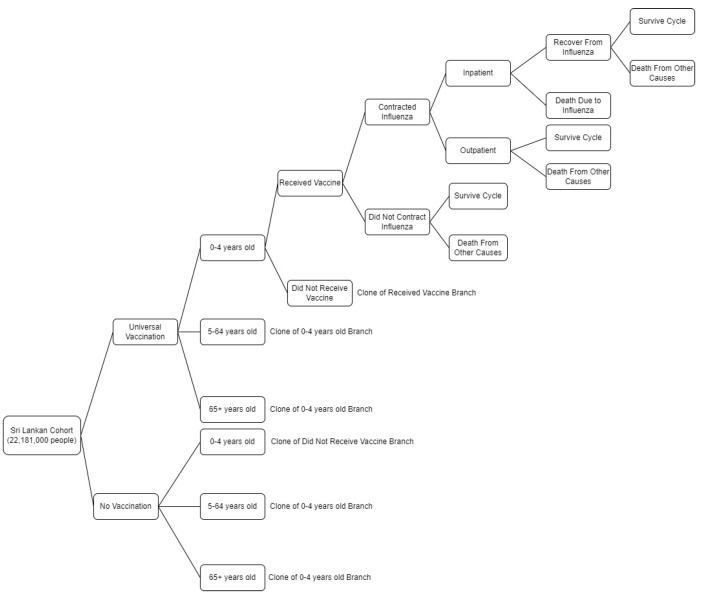
Simplified diagram of the decision model used to analyze the cost-effectiveness of a universal influenza vaccination program in Sri Lanka.

A static Markov model (Figure 1) that did not account for transmission dynamics was used for this study. The model followed a theoretical cohort of 22,181,000 Sri Lankans of all ages through two potential scenarios: universal influenza vaccination and no influenza vaccination across twelve-monthly cycles. The cohort was divided into 3 separate age groups: 0–4, 5–64, and 65+ years based on 2022 age-specific provisional mid-year population estimates [13]. 

All participants entered the model alive. They would receive an influenza vaccine based on the model arm and age-group-specific vaccine coverage rates. The participants would then contract influenza or not based on defined age-group-specific influenza incidence rates and vaccine effectiveness. Because the influenza data used for the model only included those who sought medical care, potential unreported influenza cases were not considered within the model. If participants were not hospitalized, they were automatically assumed to receive care in the outpatient setting. Once participants received inpatient or outpatient care, they would survive the infection or die. Because we worked under the assumption that participants could only contract influenza once per cycle, if a participant survived or did not contract influenza, they could die due to causes other than influenza or survive to the next cycle. All survivors alive at the end of each cycle were forced to move on to the next monthly transition until they died or until the twelve-month cycle concluded. TreeAge Pro software (version 2022; TreeAge, Inc, Williamstown, MA, USA) was used to develop the model and conduct all analyses.

### 2.3. Model Parameters

#### 2.3.1. Influenza Incidence

A three-step process was used to calculate influenza incidence estimates. We first obtained existing influenza-like illness (ILI) and severe acute respiratory infection (SARI) surveillance data from 2012 to 2017 and 2019 [15,16,17,18,19,20,21,22]. These years were chosen due to data availability and completeness. Using the national surveillance data, we calculated the monthly influenza-associated SARI estimates, influenza-associated ILI estimates, and influenza burden percentages. The method used to calculate influenza-associated SARI and ILI estimates came from the guidelines in *A Manual for Estimating Disease Burden Associated with Seasonal Influenza* [23]. Data on surveillance location catchment populations and age-specific estimates were not readily available, so these factors were excluded from estimates. After viewing these estimates and consulting previous influenza estimates in related literature, we decided to use only the calculated monthly influenza burden percentages. Monthly influenza burden percentages were multiplied by Sri Lankan annual influenza episode estimates to calculate each month’s influenza burden [4]. Due to the limited age-specific data, these monthly estimates were multiplied by the age-group-specific percentage of influenza medical visits from the United States during the 2021 to 2022 season [24]. Monthly percentage calculations for influenza episodes can be found in Table S1. 

To calculate inpatient monthly influenza estimates, we followed the same process previously discussed for monthly influenza burden; however, we only used national SARI surveillance data. Monthly influenza inpatient percentages were multiplied by annual influenza hospitalization estimates and the calculated age-group-specific rates of influenza hospitalization from Sri Lanka’s 2019 indoor morbidity and mortality report [4,25]. Monthly percentage calculations for overall inpatient influenza episodes can be found in Table S2. Because our model did not consider individuals who did not seek care, influenza-associated outpatient estimates were calculated by subtracting monthly hospitalizations from monthly episode estimates. Calculated annual rates for overall and hospitalized influenza by age group can be found in Table 1.

For those who received a vaccine within the vaccinated model arm, the influenza infection rate was calculated with the following formula:

R(t)_Vaccination_ = R(t)_No Vaccination_ × (1 − VE),

where R(t)_No Vaccination_ is the rate of influenza infection in unvaccinated populations, and VE is the age-group-specific vaccine effectiveness. 

#### 2.3.2. Mortality

A previously published estimate of 1.2 per 100,000 was used for annual influenza-related mortality [4]. Due to the limited available data regarding influenza-related mortality in Sri Lanka, we assumed that influenza-related mortality trends would closely follow influenza-related hospitalization trends. We multiplied the annual estimate by the monthly percentage of influenza-related hospitalizations (Table S2) we previously calculated. To make these estimates fit within our age groups, we multiplied the overall monthly estimate by the age-group percentages of estimated influenza-associated annual respiratory deaths for India from 2010 to 2013 [26]. 

Annual mortality from other causes for each age group were calculated from the reported deaths from each age group and the group’s population [13,27]. We divided the annual mortalities for each age group by 12 months and then subtracted the number of influenza deaths from each of the months to obtain the monthly deaths from other causes for each age group. The age-specific annual influenza-related mortality and mortality from other causes rates used for the model can be found in Table 1.

#### 2.3.3. Vaccine Coverage and Effectiveness

All vaccine model parameters can be found in Table 1. We used a variety of sources to estimate the age-specific vaccine coverage rates. For vaccine coverage in the 0–4 year age group, a uniform distribution was used with the lower end being an estimate (23.00%) given by the WHO for publicly funded influenza vaccination coverage in LMIC tropical countries and an upper estimate of 100.00% due to Sri Lanka’s vaccination rate for the newly added human papillomavirus (HPV) vaccine [28,29]. An estimate for the overall influenza vaccination uptake by the general population in Asia (14.90% [0.80%–45.00%]) was used for the vaccine coverage rate in the 5-to-64-years-old age group [30]. Finally, for the 65+ year age group, we used a WHO estimate (14.00%–41.00%) for elderly influenza vaccination coverage in LMICs with publicly funded immunization programs [28]. 

For this study, we used vaccine effectiveness (VE) estimates for trivalent inactivated influenza vaccines against laboratory-confirmed influenza for each age group. The 0–4 year age group VE estimate was derived from the adjusted VE estimate (53.4% [25.30–70.50%]) from reverse transcription-polymerase chain reaction (RT-PCR)-confirmed influenza in children 6–59 months old in Korea [31]. Due to no data being found for the 5–64 year age group, we used a pooled VE (59.00% [51.00–67.00%]) from 8 randomized controlled trials spanning 9 influenza seasons for 18–64 year olds [32]. An estimate of 49.0% (33.00–62.00%) was used for the 65+ year age group from a 2013 study that used data from estimates from over 2.5 million elderly participants [33], which was used in a similar cost-effectiveness study comparing trivalent (TIV) and quadrivalent (QIV) influenza vaccination for the elderly population in China [34].

**Table 1:** Population, epidemiological, and vaccine parameters used in the model.

Parameter	Base Case	Range	Distribution	Source
Percentage of Population by Age Group			Beta	[13]
0–4	8.59%	6.87–10.31%		
5–64	83.58%	80.29–86.86%		
65+	7.84%	6.27–9.40%		
Annual Influenza Incidence Rate per 100,000			Beta	Calculation
0–4	2419.1453			
5–64	1389.2567			
65+	1074.5235			
Annual Influenza Hospitalization Rate per 100,000			Beta	Calculation
0–4	81.1363			
5–64	20.9187			
65+	14.6561			
Annual Influenza Death Rate per 100,000			Beta	Calculation
0–4	2.7703			
5–64	0.2802			
65+	9.2901			
Annual Mortality Rate from Other Causes per 100,000			Beta	Calculation
0–4	155.9699			
5–64	232.6198			
65+	4923.9090			
Vaccine Coverage by Age Group				
0–4	61.50%	23.00–100.00%	Uniform	[28,29]
5–64	14.90%	0.80–45.00%	Beta	[30]
65+	27.50%	14.00–41.00%	Uniform	[28]
Vaccine Effectiveness Against Lab-Confirmed Influenza by Age Group				
0–4	53.40%	25.30–70.50%	PERT	[31]
5–64	59.00%	51.00–67.00%	PERT	[32]
65+	49.00%	33.00–62.00%	Triangular	[33]

#### 2.3.4. Cost Parameters

We utilized cost data from prior literature and local corporations. All costs in Sri Lankan Rupees (Rs.) were adjusted to 2022 values using annual inflation rates and then exchanged to United States dollars (USD) (Rs. 241.36/1 USD) [35]. Additionally, all costs were discounted 5.00% (4.00–6.00%) [36]. All cost parameters can be found in Table 2. 

The model’s direct medical cost included costs directly related to the patient seeking care, such as medical visit/stay cost, commonly prescribed prescriptions, and transportation. All medications were identified from previous research and verified by medical professionals with prior experience in Sri Lanka [37,38,39]. Prescription costs were based on standard dosages. The Sri Lankan mean birth weight (2.9 kg) and 5-year-old weight from India (17 kg) were used to obtain prescription dosages and expenses for the upper and lower estimates of the 0–4 year age group and the lower limit for the 5–64 year age group [40,41]. 

For hospitalized individuals, we included costs for transportation (Rs. 101.85, USD 0.42) [42], medical stay, and a prescription for oseltamivir. Medical stay costs were calculated using the hospital cost per day for primary (Rs. 3543.17, USD 14.68) and tertiary (Rs. 4776.42, USD 19.79) hospitals [43], and an average hospital stay length of 3.7 days [44]. The prescription cost for oseltamivir was calculated by multiplying the wholesale price per pill of an oseltamivir 75 mg capsule (Rs. 254.91, USD 1.06) and the average pill count for a standard prescription using CDC child weight and adult dosage recommendations for oral oseltamivir treatment for 5 days (1.16–10 pills) [45,46]. 

For outpatient individuals, we included costs for transportation (Rs. 101.85, USD 0.42) [42], medical visit (Rs. 625.12, USD 2.59), and a prescription for amoxicillin, paracetamol, and chlorpheniramine. Prescription costs were calculated using the price per 500 mg capsule of amoxicillin (Rs. 5.73, USD 0.02), 500 mg tablet of paracetamol (Rs. 1.22, USD 0.00), and 500 mg tablet of chlorpheniramine (Rs. 0.16, USD 0.00) multiplied by the standard child weight and adult dosage recommendations for each [45,47,48,49]. Previous studies conducted in Southern Sri Lanka identified that the average influenza patient treated in the outpatient setting receives a mean of three prescriptions, with the above prescriptions being the most commonly administered [38]. All outpatient prescriptions were calculated based on a three-day prescription due to Sri Lankan doctors only being allowed to prescribe three-day dosages within the outpatient public hospital setting. 

In addition to direct costs, we also considered the indirect costs one might acquire if they contracted influenza in Sri Lanka. Included in the indirect costs were income and productivity losses due to work absenteeism and presenteeism. The cost of absenteeism and presenteeism was calculated by multiplying the average number of days of total productivity loss (6.95) and the daily per capita income (Rs. 541.82, USD 2.24) [50]. The daily per capita income was calculated by dividing the per capita monthly income (Rs. 16,491.32, USD 68.33) by the average number of days per month (30.44) [51]. 

The cost for vaccination was defined as the cost of the vaccine per dose, a disposable 2 mL syringe without a needle (Rs. 5.32, USD 0.02) [52] and a 22-gauge disposable needle (Rs. 3.60, USD 0.01) [52]. The per-dose vaccine cost was retrieved from the average price per dose of the 2019 pediatric and adult seasonal influenza TIV southern hemisphere formulation for both Korean and French origins released by the WHO and the Pan American Health Organization (PAHO) as a part of the expanded program of immunization vaccine prices for 2019 [53].

**Table 2:** Parameters used to calculate costs in the model.

Parameter	Base Case *	Range	Distribution	Source
Average Weight (kg)				
Newborn	2.9			[40]
5 Year Old	17			[41]
Inpatient Direct Medical Costs				
Medical Visits per Day	4159.80 (17.23)	3543.17–4776.42 (14.68–19.79)	Uniform	[43]
Hospital Duration (days)	3.7	2.96–4.44	PERT	[44]
Oseltamivir 75 mg Capsule	254.91 (1.06)	203.93–305.89 (0.84–1.27)	Gamma	[45]
Transportation	101.85 (0.42)	52.38–174.61 (0.22–0.72)	Gamma	[42]
Outpatient Direct Medical Costs				
Medical Visit	625.12 (2.59)	506.99–743.25 (2.10–3.08)	Uniform	[43]
Amoxicillin 500 mg Capsule	5.73 (0.02)	5.45–5.91 (0.02–0.02)	Gamma	[45]
Paracetamol 500 mg Tablet	1.22 (0.00)	0.97–1.46 (0.00–0.01)	Gamma	[45]
Chlorpheniramine 500 mg Tablet	0.16 (0.00)	0.13–0.19 (0.00–0.00)	Gamma	[45]
Transportation	101.85 (0.42)	52.38–174.61 (0.22–0.72)	Gamma	[42]
Indirect Medical Costs				
Days Lost to Absenteeism and Presenteeism	6.95	0–34.05	Gamma	[50]
Daily per Capita Income	541.82 (2.24)	425.67–638.50 (1.76–2.65)	PERT	Calculation
Vaccine Costs				
Vaccine per Dose				[53]
0–4 year age group	248.38 (1.03)	224.78–271.99 (0.93–1.13)	Uniform	
5–64 year age group	450.06 (1.86)	224.78–675.35 (0.93–2.80)	Uniform	
65+ year age group	562.45 (2.33)	449.55–675.35 (1.86–2.80)	Uniform	
Disposable 22 G Needle	3.60 (0.01)	2.88–4.71 (0.01–0.02)	Gamma	[52]
Disposable 2 m Syringe w/o needle	5.32 (0.02)	4.25–6.43 (0.02–0.03)	Gamma	[52]

* All costs are presented as Sri Lankan Rupees (United States Dollars). 1 USD = Rs. 241.36.

#### 2.3.5. Disability-Adjusted life Years

We estimated the disability-adjusted life years (DALYs) averted by implementing a universal vaccination program compared to the current no influenza vaccination policy context. This analysis accounted for years of life lost (YLLs) due to premature death from influenza and years lived with disability (YLDs) from contracting influenza. The following equation was used to obtain the total DALYs for each month:

DALY = YLL + YLD

Additionally, all health outcome values were discounted by 5.00% (4.00–6.00%) [36]. 

YLL estimates due to premature death were calculated for each month. Monthly YLL estimates were calculated by multiplying our calculated monthly influenza deaths in each age group (Table 3) by the weighted life expectancy for that age group. Weighted age group life expectancies were found using age-specific national population and life expectancy estimates [54,55]. YLD estimates from contracting influenza were derived from two previously defined disability weight estimates. For hospitalized individuals, we used an estimate for severe lower respiratory tract infection (0.133), while we used an estimate for moderate lower respiratory tract infection (0.051) for outpatient individuals [56].

### 2.4. Cost-Effectiveness Analysis and Sensitivity Analysis

One model arm was considered dominated when it offered lower DALYs averted and was more expensive than was the other option. When universal vaccination was deemed to be dominant (higher DALY averted) at a higher cost compared to no vaccination, the following equation was used to calculate the incremental cost-effectiveness ratio (ICER) of the universal vaccination:

ICER = (Cost_Vaccination_ − Cost_No Vaccination_)/(DALY_No Vaccination_ − DALY_Vaccination_).

One model arm was considered cost-effective if the ICER was below a three-fold gross domestic product (GDP) per capita per DALY averted limit and highly cost-effective if below a one-fold GDP per capita per DALY averted limit [57]. The 2022 GDP per capita at current prices (Rs. 892,672.48, USD 3698.56) for Sri Lanka was used [35]. With this data, the willingness-to-pay (WTP) threshold used in this study was defined as Rs. 2,678,017.44 (USD 11,095.53) per DALY averted (three-fold of GDP per capita at market price). The highly cost-effective threshold was defined as less than Rs. 892,672.48 (USD 3698.56) per DALY averted (one-fold the GDP per capita at market price). 

We performed probabilistic sensitivity analyses to explore the parameter influence on the resulting incremental cost-effectiveness ratios (ICERs). Probabilistic sensitivity analyses were completed by using Monte Carlo sampling with distributions (95% CI or ± 20% of base-case values) for all model parameters and 10,000 simulations being run and then by calculating the mean and 95% CI for the ICERs based on these 10,000 simulations. In addition, we completed one-way sensitivity analyses for all parameters over variable ranges (95% CI or ±20% of base-case values) to identify any influential parameters on the base-case results. 

### 2.5. Budget Impact Analysis

A budget impact analysis (BIA) was calculated to project the financial burden if Sri Lanka introduced a universal influenza vaccination program compared to their current no-vaccination policy for one year. The total budget of the vaccination program was calculated by multiplying the vaccination costs (Table 2) by the estimated number of people that would receive the vaccine in each age group, which were calculated using the estimated vaccine coverage rates (Table 1). The treatment costs averted due to the influenza vaccine were calculated to obtain the net incremental cost for the program. Sri Lanka’s healthcare spending budget (Rs. 751.46 billion; USD 3.11 billion) was calculated by multiplying Sri Lanka’s 2018 total health expenditure as a share of GDP (3.8%) by their 2022 GDP at current prices (Rs. 19,775.24 billion; USD 81.93 billion) [35,58]. No discounting was used for any costs or health outcomes within the BIA.

## 3. Results

### 3.1. Base-Case Analysis

The estimated number of individuals who contracted influenza or who died or were hospitalized from it and the costs for each age group are shown in Table 4, along with the ICER for the base case. Under the base-case assumptions, vaccinations resulted in an overall decrease in influenza cases, hospitalizations, and death. Vaccinations would be expected to reduce the number of influenza episodes by 20,711, hospitalizations by 439, and deaths by 21 compared to no vaccination in one year. Universal TIV vaccination was cost-effective (ICER = 874,890.55 Rs/DALY averted; 3624.84 USD/DALY averted) at the three times and one times the GDP WTP thresholds. 

### 3.2. Budget Impact Analysis

The BIA results for TIV vaccine implementation can be found in Table 5. The annual cost of implementing a universal vaccination policy in Sri Lanka was estimated at Rs. 2,550,805,095.84 (USD 10,568,466.59). With our estimates, Sri Lanka spends roughly Rs. 809,097,083.59 (USD 3,352,241.81) annually on influenza. With our base case analysis, Sri Lanka would advert 20,643 cases and 20 deaths in one year and have a net cost increase of Rs. 1,741,708,012.25 (USD 7,216,224.78), which is equal to 0.23% of Sri Lanka’s estimated healthcare spending. If Sri Lanka only vaccinated the 0-to-4 year age group and the 65+ year age group, 8551 cases and 18 deaths would be adverted and there would be a net cost increase of Rs. 531,769,189.25 (USD 2,203,220.04), 0.07% of Sri Lanka’s estimated healthcare spending, in one year.

### 3.3. Sensitivity Analyses

**Figure 2:**
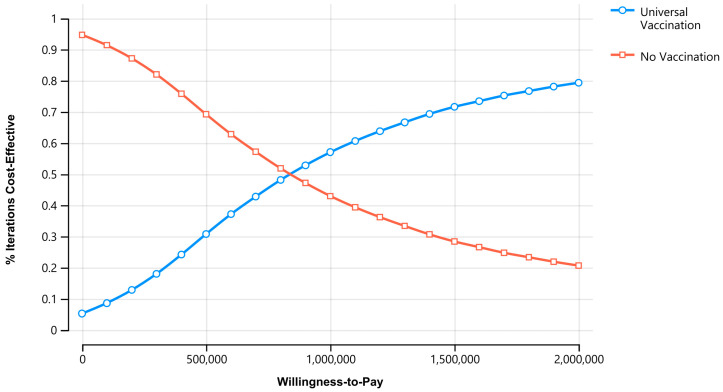
Acceptability curve for universal influenza vaccination vs. no vaccination. Universal vaccination became more acceptable than no vaccination at 874,890.55 Rs/DALY averted (3624.84 USD/DALY averted).

Figure 2 shows the cost-effectiveness acceptability curve for universal influenza vaccination versus no vaccination. At the cost-effective WTP threshold (3× GDP per capita), universal vaccination was favored 85.16% of the time and 52.58% of the time at the highly cost-effectiveness WTP threshold (1× GDP per capita). Universal vaccination became cost-effective at approximately 98.01% of Sri Lanka’s 2022 GDP per capita (ICER = 874,890.55 Rs/DALY averted; 3624.84 USD/DALY averted). 

**Figure 3:**
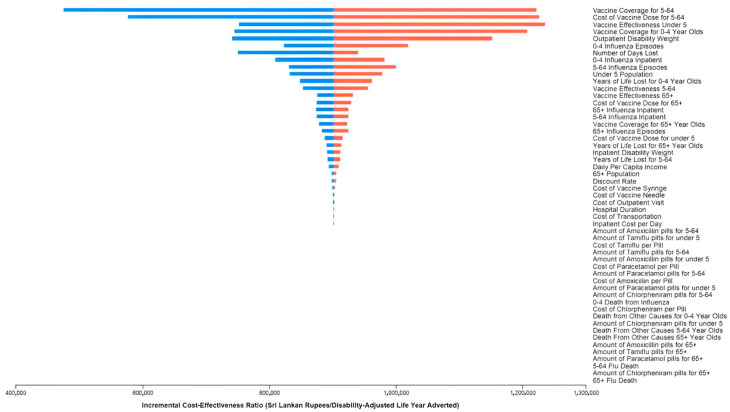
One-way sensitivity analysis results. The incremental cost-effectiveness ratio (ICER) is presented in Sri Lankan Rupees (Rs.). The figure is centered at the expected ICER value (874,890.55 Rs/DALY averted; 3624.84 USD/DALY averted). Each variable shows the maximum and minimum values for the range that we have used. The blue bar indicates when the ICER will be below the expected ICER value for each variable, and the red bar shows when the ICER will be above the expected ICER value for each variable. No value for a variable within our estimated ranges resulted in ICERs above Rs. 1,300,000 (USD 5386.15) per DALY adverted.

Figure 3 shows the one-way sensitivity analysis for vaccination. Results were most sensitive to the vaccine coverage in the 5–64-years-old age group, the cost of the influenza vaccine dose in the 5–64-years-old age group, vaccine effectiveness in the under-5-years-old age group, and the vaccine coverage in the under-5-years-old age group. No value for a variable within our estimated ranges resulted in ICERs above Rs. 1,300,000 (USD 5386.15) per DALY adverted, which is greater than the highly cost-effective threshold of Rs. 829,672.48 (USD 3698.56) but below the cost-effective WTP threshold of Rs. 2,678,017.44 (USD 11,095.53) per DALY averted. 

## 4. Discussion

This study estimated the disease burden of influenza using previous estimates and Sri Lankan national data and analyzed the cost-effectiveness of influenza vaccination at a national level. We conducted a cost-effectiveness analysis for the Sri Lankan population to determine the priority of influenza vaccine implementation. This study showed that including influenza vaccination in the NIP could represent good value for money in Sri Lanka despite the increase in cost it would require to implement. 

Due to limited access to data, we made a few assumptions within the model. The first assumption was that once participants developed immunity against influenza by surviving it, they would not contract it again within the same monthly cycle. We also assumed that influenza mortality followed a similar monthly pattern to monthly hospitalizations. Adverse events related to vaccination, influenza-related complications, herd immunity effects, and the potential increase in vaccine program costs were not considered. 

We are unaware of other studies evaluating the cost-effectiveness of influenza vaccination in Sri Lanka. While there have been cost-effectiveness studies evaluating influenza vaccination programs, most are in high-income settings, with fewer in LMICs [59]. Cost-effectiveness studies conducted in LMICs evaluating influenza vaccinations in different populations have found that influenza vaccines are generally cost-effective [59,60]. Studies from high-income countries have also found that influenza vaccinations usually are cost-effective in diverse populations [61,62,63,64,65]. 

This study does have limitations. First, this study used a static model and not a dynamic model, which means the model cannot consider potentially important factors, such as herd immunity effects. By using a static model, the study does, however, provide a conservative estimate for the cost-effectiveness of influenza vaccinations. Second, our model only considers the effects of universal vaccination in one year and does not consider any rollover effects in future years. The effectiveness of influenza vaccines has been found to decrease with time [66]. However, since vaccination was cost-effective when only considering the effects on one year, adding multiple years would likely prove more cost-effective due to rollover effects when citizens are vaccinated later in the year. Third, the input data used for the model might have underestimated the disease burden of influenza. Patients with mild infections might not seek medical care. However, since the study goal was to verify an influenza vaccination program’s cost-effectiveness from a societal perspective, we believe this analysis serves well as a conservative estimate. Since data were limited regarding influenza epidemiology and costs for Sri Lanka specifically, we were required to use estimations from other countries or calculate estimates. This might have introduced uncertainty into the model. To address this potential issue, we ran probabilistic and one-way sensitivity analyses on all variables within the model. Finally, this study did not deliberately account for the feasibility of the policy. Many unobserved variables were not included in the model, including staffing, storage capacity, additional medical treatments and diagnostic tests, and the indirect costs of caregivers. Since we wanted to estimate the local context within Sri Lanka, these costs were excluded due to uncertainty, limited data, or no routine usage. As a result, the results should be interpreted cautiously; however, we believe they provide a reasonable conservative estimate. 

Despite these limitations, this study has the advantage of reflecting the local context. A limited number of studies have been conducted to investigate seasonal influenza in Sri Lanka [4,10,11,12,37,38,39,42,67]. The contribution of previous studies was the estimation of influenza disease burden and available treatment options and costs. However, some studies have been limited to mainly Southern Sri Lanka [11,37,38,42,67]. Other studies, such as those by Rafeek et al. and Jayasinghe et al. [10,12], describe Sri Lankan influenza trends on a national level. The Global Burden of Disease Study 2017 provided an influenza disease burden estimate at the national level [4]; however, this estimate is only an annual estimate. Our study addressed these limitations by developing a method to estimate the monthly influenza disease burden at a national level. 

## 5. Conclusions

This study showed that including influenza vaccinations in the NIP would be highly cost-effective in Sri Lanka and suggests that vaccine implementation should be considered. However, large-scale national studies with improved data regarding current influenza epidemiology and immunology and economic costs are needed to better inform estimates and determine the impacts of such a policy.

## Figures and Tables

**Table 3:** Utility parameters used in the model.

Parameter	Base Case	Range	Distribution	Source
Disability Weight per Influenza Episode			Normal	[56]
Nonhospitalized	0.051	0.032–0.074		
Hospitalized	0.133	0.088–0.190		
Years of Life Lost by Age Group			PERT	Calculation
0–4 year age group	76.44	61.15–91.72		
5–64 year age group	48.67	38.93–58.40		
65+ year age group	12.53	10.02–15.03		

**Table 4:** Discounted base-case results across one year.

Parameters	No Vaccination	Universal Vaccination	Universal Vaccination vs. No Vaccination
Health Outcomes			
Influenza Cases	167,847	147,137	−20,710
0–4 year olds	23,002	15,696	−7306
5–64 year olds	135,245	123,103	−12,142
65+ year olds	9600	8338	−1262
Hospitalizations	2867	2429	−438
0–4 year olds	774	529	−245
5–64 year olds	1967	1790	−177
65+ year olds	126	110	−16
Outpatients	164,980	144,708	−20,272
0–4 year olds	22,228	15,167	−7061
5–64 year olds	133,278	121,313	−11,965
65+ year olds	9474	8228	−1246
Deaths from Influenza	130	110	−20
0–4 year olds	26	18	−8
5–64 year olds	25	23	−2
65+ year olds	79	69	−10
DALYs	13,072.89	11,105.06	−2013.63
Total Cost ^+^	793,871,356.06 (3289,158.75)	2,555,577,215.23 (10,588,238.38)	1,761,705,859.18 (7,299,079.63)
Incremental Cost per DALY adverted ^+^			874,890.55 (3624.84)

* DALY = disability-adjusted life year. ^+^ Costs presented in Sri Lanka Rupees (United States Dollars). 1 USD = Rs. 241.36.

**Table 5:** Budget impact analysis across one year with undiscounted costs and health outcomes.

	0–4 Year Olds	5–64 Year Olds	65+ Year Olds	0–4 Year Olds and 65+ Year Olds	Total
No Vaccination					
Population Size	1,905,000	18,538,000	1,738,000	3,643,000	22,181,000
Number of Influenza Cases	22,997	135,246	9603	32,600	167,846
Indirect Medical Costs	86,597,692.12 (358,790.57)	509,287,654.66 (2,110,074.80)	36,160,134.37 (149,818.26)	36,160,134.37 (508,608.83)	36,160,134.37 (2,618,683.63)
Number of Hospitalizations	774	1966	126	900	2866
Hospitalization Costs	12,706,505.31 (52,645.45)	34,477,676.51 (142,847.52)	2,282,455.85 (9456.65)	14,988,961.16 (62,102.09)	49,466,637.67 (204,949.61)
Number of Outpatient Visits	22,223	133,280	9477	31,700	164,980
Outpatient Costs	16,584,539.29 (68,712.87)	103,477,649.37 (428,727.42)	7,522,776.11 (31,168.28)	24,107,315.40 (99,881.15)	127,584,964.77 (528,608.57)
Number of Deaths	26	25	79	105	130
Total Costs	115,888,736.73 (480,148.89)	647,242,980.53 (2,681,649.74)	45,965,366.33 (190,443.18)	161,854,103.06 (670,592.07)	809,097,083.59 (3,352,241.81)
**Universal Vaccination**					
Number of Influenza Cases	15,710	123,154	8339	24,049	147,203
Indirect Medical Costs	59,159,420.15 (245,108.64)	463,756,368.98 (1,921,430.10)	31,400,951.48 (130,100.06)	90,560,371.63 (375,208.70)	554,316,740.61 (2,296,638.80)
Number of Hospitalizations	529	1791	110	639	2430
Hospitalization Costs	8,683,201.42 (35,976.14)	31,412,256.45 (130,146.90)	1,982,493.31 (8213.84)	10,665,694.73 (44,189.98)	42,077,951.18 (174,336.89)
Number of Outpatient Visits	15,181	121,363	8229	23,410	144,773
Outpatient Costs	11,329,643.81 (46,940.85)	94,225,800.08 (390,395.26)	6,532,653.44 (27,066.02)	17,862,297.26 (74,006.87)	112,088,097.34 (464,402.13)
Number of Vaccines Given	1,171,575	2,762,162	477,950	1,649,525	4,411,687
Vaccination Costs	301,447,885.94 (1,248,955.44)	1,267,787,378.03 (5,252,682.21)	273,087,042.74 (1,131,451.12)	574,534,928.68 (2,380,406.57)	1,842,322,306.71 (7,633,088.77)
Number of Deaths	18	23	69	87	110
Total Costs	380,620,151.33 (1,576,981.07)	1,857,181,803.53 (7,694,654.47)	313,003,140.98 (1,296,831.04)	693,623,292.30 (2,875,549.76)	2,550,805,095.84 (10,568,466.59)
**No Vaccination v. Universal Vaccination**					
Number of Influenza Cases	7287	12,092	1264	8551	20,643
Indirect Costs	27,438,271.97 (113,681.94)	45,531,285.68 (188,644.70)	4,759,182.89 (19,718.19)	32,197,454.86 (133,400.13)	77,728,740.54 (322,044.83)
Number of Hospitalizations	245	175	16	261	436
Hospitalization Costs	4,023,303.89 (16,669.31)	3,065,420.06 (12,700.61)	299,962.54 (1,242.80)	4,323,266.43 (17,912.11)	7,388,686.49 (30,612.72)
Number of Outpatient Visits	7042	11,917	1248	8290	20,207
Outpatient Costs	5,254,895.48 (21,772.02)	9,251,849.29 (38,332.16)	990,122.66 (4,102.26)	6,245,018.14 (25,874.29)	15,496,867.43 (64,206.44)
Vaccination Costs	−301,447,885.94 (−1,248,955.44)	−1,267,787,378.03 (−5,252,682.21)	−273,087,042.74 (−1,131,451.12)	−574,534,928.68 (−2,380,406.57)	−1,842,322,306.71 (−7,633,088.77)
Number of Deaths	8	2	10	18	20
Total Costs	−264,731,414.60 (−1,096,832.18)	−1,209,938,823.00 (−5,013,004.74)	−267,037,774.65 (−1,106,387.86)	−531,769,189.25 (−2,204,957.69)	−1,741,708,012.25 (−7,216,224.78)
Total Costs as a Percentage of Health Spending	0.04%	0.16%	0.04%	0.07%	0.23%

Costs are presented in Sri Lanka Rupees (United States Dollars). 1 USD = Rs. 241.36.

## Data Availability

No new data were created in this study. Data sharing is not applicable to this article.

## Supplementary Materials

The following supporting information can be downloaded at https://www.mdpi.com/article/10.3390/vaccines11050932/s1: Table S1: Calculated monthly percentages for the overall influenza burden; Table S2: Calculated monthly percentages for hospitalized influenza cases.
