# Supplementary material for: Influenza Vaccination Implementation in Sri Lanka: A Cost-Effectiveness Analysis
Source: Vaccines (Basel). 2023 May 3;11(5):932. doi: 10.3390/vaccines11050932 (PMC10220975; doi:10.3390/vaccines11050932)
Supplement: Supplementary file 1 [file vaccines-2346703-supplementary.pdf › vaccines-2346703-supplementary.pdf]

Table S1. Calculated monthly percentages for the overall influenza burden.

	2012		2013		2014		2015		2016		2017		2019		Average % of Annual Cases for 2012-2019
	ILI + SARI cases	% of Annual Cases	ILI + SARI cases	% of Annual Cases	ILI + SARI cases	% of Annual Cases	ILI + SARI cases	% of Annual Cases	ILI + SARI cases	% of Annual Cases	ILI + SARI cases	% of Annual Cases	ILI + SARI cases	% of Annual Cases	
**Jan**	11,446	13.75%	6,229	6.69%	7,202	20.50%	4,453	10.27%	10,834	9.72%	5,789	6.45%	6,820	6.29%	10.53%
**Feb**	8,431	10.13%	8,134	8.74%	4,589	13.06%	3,982	9.19%	7,853	7.05%	7,842	8.74%	7,139	6.59%	9.07%
**Mar**	6,992	8.40%	8,690	9.34%	1,086	3.09%	3,935	9.08%	9,110	8.17%	12,433	13.85%	11,033	10.18%	8.87%
**Apr**	5,557	6.68%	6,753	7.26%	785	2.23%	2,629	6.06%	10,650	9.56%	7,977	8.89%	6,406	5.91%	6.65%
**May**	4,303	5.17%	10,522	11.31%	3,632	10.34%	2,488	5.74%	8,746	7.85%	6,811	7.59%	5,876	5.42%	7.63%
**Jun**	8,110	9.74%	8,822	9.48%	3,194	9.09%	2,877	6.64%	9,605	8.62%	7,559	8.42%	7,229	6.67%	8.38%
**Jul**	7,869	9.45%	8,857	9.52%	4,186	11.91%	4,074	9.40%	14,260	12.79%	5,407	6.02%	8,840	8.15%	9.61%
**Aug**	6,543	7.86%	7,198	7.74%	2,877	8.19%	4,742	10.94%	10,216	9.17%	4,638	5.17%	7,920	7.31%	8.05%
**Sep**	5,527	6.64%	6,443	6.92%	2,249	6.40%	4,025	9.28%	9,922	8.90%	8,218	9.15%	7,111	6.56%	7.70%
**Oct**	5,414	6.50%	7,250	7.79%	2,351	6.69%	4,062	9.37%	8,341	7.48%	7,324	8.16%	9,180	8.47%	7.78%
**Nov**	6,590	7.92%	8,068	8.67%	1,261	3.59%	4,042	9.32%	4,977	4.47%	6,609	7.36%	16,728	15.43%	8.11%
**Dec**	6,458	7.76%	6,088	6.54%	1,721	4.90%	2,041	4.71%	6,944	6.23%	9,163	10.21%	14,118	13.02%	7.62%
**Sum**	83,240	100.00%	93,054	100.00%	35,133	100.00%	43,350	100.00%	111,458	100.00%	89,770	100.00%	108,400	100.00%	100.00%

* ILI= influenza-like illness; SARI= Severe acute respiratory infection

Table S2. Calculated monthly percentages for hospitalized influenza cases.

	2012		2013		2014		2015		2016		2017		2019		Average % of Annual Cases for 2012-2019
	SARI cases	% of Annual Cases	SARI cases	% of Annual Cases	SARI cases	% of Annual Cases	SARI cases	% of Annual Cases	SARI cases	% of Annual Cases	SARI cases	% of Annual Cases	SARI cases	% of Annual Cases	
**Jan**	181	7.02%	87	2.47%	277	13.17%	60	2.01%	43	5.27%	108	7.95%	267	6.17%	6.29%
**Feb**	83	3.22%	153	4.34%	402	19.11%	401	13.40%	61	7.48%	196	14.42%	354	8.18%	10.02%
**Mar**	200	7.75%	498	14.13%	126	5.99%	137	4.58%	60	7.35%	324	23.84%	568	13.13%	10.97%
**Apr**	193	7.48%	519	14.73%	95	4.52%	166	5.55%	61	7.48%	228	16.78%	442	10.21%	9.53%
**May**	165	6.40%	444	12.60%	387	18.39%	115	3.84%	33	4.04%	78	5.74%	316	7.30%	8.33%
**Jun**	173	6.71%	397	11.27%	293	13.93%	204	6.82%	81	9.93%	55	4.05%	183	4.23%	8.13%
**Jul**	267	10.35%	404	11.46%	236	11.22%	859	28.71%	126	15.44%	31	2.28%	309	7.14%	12.37%
**Aug**	314	12.17%	253	7.18%	120	5.70%	390	13.03%	74	9.07%	11	0.81%	261	6.03%	7.71%
**Sep**	259	10.04%	248	7.04%	40	1.90%	220	7.35%	82	10.05%	44	3.24%	173	4.00%	6.23%
**Oct**	301	11.67%	235	6.67%	75	3.56%	356	11.90%	55	6.74%	33	2.43%	389	8.99%	7.42%
**Nov**	367	14.22%	231	6.56%	32	1.52%	32	1.07%	43	5.27%	63	4.64%	510	11.79%	6.44%
**Dec**	77	2.98%	55	1.56%	21	1.00%	52	1.74%	97	11.89%	188	13.83%	555	12.83%	6.55%
**Sum**	2,580	100.00%	3,524	100.00%	2,104	100.00%	2,992	100.00%	816	100.00%	1,359	100.00%	4,327	100.00%	100.00%

* SARI= Severe acute respiratory infection
